# 79664 Complement Driven Auto-Reactive Antibodies in Lung Transplantation

**DOI:** 10.1017/cts.2021.449

**Published:** 2021-03-30

**Authors:** Alexander McQuiston, Changhai Li, Kunal Patel, Zhenxiao Tu, Dianna Nord, Satish Nadig, Stephen Tomlinson, Carl Atkinson

**Affiliations:** 1 Medical University of South Carolina; 2 University of Florida

## Abstract

ABSTRACT IMPACT: Our work unveils a novel mechanism of ischemia repurfusion injury driven by pre-existing autoimmunity following lung transplant and a potential therapeutic strategy for blocking complement-dependent injury thereby reducing risk of lung transplant rejection. OBJECTIVES/GOALS: Our goal was to determine if pre-existing autoimmune autoantibodies, such as those resulting from cigarette smoke (CS), contribute to graft rejection in lung transplantation (LTx) and if autoreactive-mediated graft injury is complement-dependent. METHODS/STUDY POPULATION: For in vivo experiments, we utilized our emphysema mouse model. Briefly, eight-week-old C57BL/6J mice are exposed to 3R4F reference cigarette smoke 5 hours per day, 5 days a week for 6 months. Upon completion, cigarette smoked (CS) mice and control (NS) mice received syngeneic orthotopic left-lung transplant from age-matched C57BL/6J donors. To determine if pre-existing autoreactivity mediated graft injury was complement-dependent we treated CS-LTx mice with a novel, bifunctional complement inhibitor. Autoantibody levels were measured by ELISA and lung injury was assessed by blinded histopathological analyses. Complement inhibition was verified by immunofluorescence. RESULTS/ANTICIPATED RESULTS: We found that CS-exposure leads to production of autoreactive antibodies towards extracellular matrix (ECM) components and contributes to graft injury. Interestingly, LTx into CS exposed mice further increased de-novo ECM autoantibody development. Lastly, treatment with our novel, bifunctional complement inhibitor blocked autoantibody spreading and significantly reduced graft rejection. DISCUSSION/SIGNIFICANCE OF FINDINGS: These data demonstrate that smoking induces pre-LTx autoreactivity to ECM proteins that promotes graft injury following LTx. Furthermore, complement inhibition reduces autoantibody production and protects the graft from injury.

